# Neurodevelopmental Effects of Perinatal TCDD Exposure Differ from Those of Other PCDD/Fs in Vietnamese Children Living near the Former US Air Base in Da Nang, Vietnam

**DOI:** 10.3390/toxics11020103

**Published:** 2023-01-21

**Authors:** Nghi Ngoc Tran, Tai Pham-The, Thao Ngoc Pham, Hoa Thi Vu, Khue Ngoc Luong, Muneko Nishijo

**Affiliations:** 1 Ministry of Health, Vietnam Government, Hanoi 10000, Vietnam; 2 Faculty of Medicine, Vietnam Military Medical University, Hanoi 10000, Vietnam; 3 Department of Public Health, Kanazawa Medial University, Uchinada 252-0815, Ishikawa, Japan

**Keywords:** dioxins, 2,3,7,8-tetrachlorodibenzo-p-dioxin (TCDD), neurodevelopment, birth cohort study, children, Vietnam

## Abstract

This study reports that children exposed to 2,3,7,8-tetra-chlorodibenzo-p-dioxin (TCDD), the major toxin in Agent Orange, from the breast milk of mothers residing near the former Da Nang US air base in Vietnam may have specific alterations in higher brain functions, resulting in social and communication deficits, including autism spectrum disorder (ASD). After the age of 8 years, girls with high TCDD showed increased attention deficit hyperactivity disorder (ADHD)-like behaviors and altered mirror neuron activity, which is often observed in children with ASD. However, no significant relationship between autistic traits and toxic equivalency values of polychlorinated dibenzodioxins and polychlorinated dibenzofurans (TEQ-PCDD/Fs) was found in these children. Notably, boys with high levels of TEQ-PCDD/Fs showed poor language and motor development in the first 3 years of life, although boys with high TCDD levels did not. However, at 8 years of age, boys with high TCDD showed reading learning difficulties, a neurodevelopmental disorder. These findings suggest that perinatal TCDD exposure impacts social–emotional cognitive functions, leading to sex-specific neurodevelopmental disorders—learning difficulty in boys and ADHD in girls. Future studies with a greater number of children exposed to high levels of TCDD are necessary to estimate the threshold values for neurodevelopmental effects.

## 1. Introduction

Da Nang Air Base, located in central Vietnam, is a former U.S. air base contaminated with dioxins from the use of Agent Orange and other herbicides containing 2,3,7,8-tetrachlorodibenzo-p-dioxin (TCDD) during the Vietnam War. We previously measured levels of 17 polychlorinated dibenzodioxins and polychlorinated dibenzofuran (PCDD/F) congeners in the breast milk of mothers residing nearby Da Nang Air Base and found these to be three- to four-fold higher than those in the breast milk of mothers living in unsprayed areas [1]. These results suggested that environmental contamination by dioxins is still high enough to increase health risks in the residents living in hot spots of dioxin contamination, even 40 years after the end of the war.

The effects of dioxins on infant neurodevelopment have previously been examined in studies in Europe [2] and Japan [3]. We therefore followed up infants whose mother’s milk samples were examined in our survey in Da Nang [1]. Our investigation of this Da Nang birth cohort, from 4 months to 8 years of age, identified adverse effects of dioxin exposure on infant and child neurodevelopment in several age groups, using standardized test batteries and parent rating scales for different aspects of neurodevelopment [4,5,6,7,8,9,10,11,12].

In the follow-up study of the Da Nang cohort, performed when the children reached 3 years of age, we found increased autistic traits (poor social and communication abilities, such as is observed in autism spectrum disorder (ASD)) in children of both sexes, associated with increased TCDD exposure but not TEQ-PCDD/F exposure [4]. Interestingly, we found that only boys showed poor language and motor development associated with high TEQ-PCDD/F exposure (but not TCDD exposure). These results suggest that TCDD may have specific neurodevelopmental effects that differ from those of other dioxin congeners.

In boys at 5 years of age, cognitive ability, assessed with the non-verbal index (NVI) of the Kaufman Assessment Battery for Children, second edition (KABC-II), was significantly lower in the high TCDD group, whereas poor coordination movement skills, indicated by the Movement Assessment Buttery for Children, second edition (MABC-2), were observed in those with high TEQ-PCDD/F levels [9]. At the same age, girls in the high TCDD group showed higher unusual behavioral scores, indicating increased autistic behavior associated with TCDD exposure, but not TEQ-PCDD/F exposure [10]. Moreover, at 8 years of age, boys with high TCDD showed reading learning disabilities, lower language achievement test scores, and poor reading skills [11].

Here, we reviewed the results of long-term follow-up studies of birth cohorts to clarify the specific neurotoxic effects of TCDD in perinatally exposed children. We focused on the following three aspects of toxicity: (1) the stage of neurodevelopment affected by TCDD; (2) differences between sexes regarding sensitivity to TCDD; and (3) the threshold values of TCDD for significant neurodevelopmental impairments.

## 2. Materials and Methods

### 2.1. Literature Searches

At first, a search of the literature was conducted using PubMed in English to find birth cohorts followed up for a long time to investigate associations between perinatal PCDD/F exposure and neurodevelopment. The following terms were used in the search procedure: “dioxins” AND “neurodevelopment” AND “birth cohort”. We found three birth cohorts followed up for a long period of time until school age, including the Hokkaido birth cohort in Japan, the Duisburg birth cohort in Germany, and the Da Nang birth cohort in Vietnam.

The TCDD exposure levels in maternal blood were too low (0.9 pg/g lipid of the geometrical mean (GM) and 3.1 pg/g lipid of maximum) and examined within narrow ranges (the 25th value is under the detection limit and the 75th value is 1.4 pg/g lipid) [3], and therefore, no significant associations between neurodevelopment and TCDD were obtained in the follow-up studies of the Hokkaido birth cohort at 6 months [3,13], 18 months [14], 42 months [15], or 13 years of age [16]. In the follow-up studies of the Duisburg birth cohort, the authors used only TEQ-PCDD/Fs as dioxin exposure markers (13.55 of pg TEQ/g lipid of the GM and 12.45–14.75 pg TEQ/g lipid at 95% confidence intervals in blood samples) [17] and showed no association between TCDD and behavioral indices in children at 2 years [18], 6–8 years [19], or 9–10 years [17,20]. Only the Da Nang birth cohort studies that showed associations between neurodevelopmental markers and TCDD and other PCDD/F congeners in their follow-up studies until 8 years of age [4,5,6,7,8,9,10,11,12] were thus selected for the present review.

### 2.2. Profile of the Da Nang Birth Cohort

The Da Nang cohort in Vietnam consisted of 241 mother–infant pairs (137 boys and 104 girls) living in the dioxin hot spots in the Thanh Khe and Son Tra districts in Da Nang City, located within 10 km of Da Nang Air Base [1]. These mother–infant pairs were recruited for the study by obstetricians at each district hospital when admitted for delivery. The criteria for recruitment were as follows: (1) mothers who resided in the study districts for at least the duration of their pregnancy; (2) babies who were full-term and healthy at birth; and (3) mothers who had no complications during pregnancy and childbirth.

As perinatal exposure markers, the dioxin levels in the maternal breast milk were used in all follow-up studies. A breast milk sample was collected from each nursing mother 1 month after birth with the assistance of a midwife or medical worker. Approximately 10 mL of breast milk from each sample was used to quantify the levels of 17 different 2,3,7,8-substituted PCDD and PCDF congeners by the established method of analysis described in detail elsewhere [1]. The toxic equivalent factors for calculating the toxic equivalents (TEQ) of PCDDs/Fs (TEQ-PCDDs/Fs) were referenced from the WHO 2005-TEF [21]. The subjects were divided into two to four groups according to the levels of TCDD and TEQ-PCDD/Fs, with cut-off values calculated using the GM and geometrical standard deviation (GSD) of dioxin levels in the breast milk of 138 nursing mothers in unsprayed areas, as follows: GM × GSD^3^ of the TCDD level (3.5 pg/g lipid) for the high TCDD group and GM × GSD^4^ of the TEQ-PCDD/F level for the high TEQ-PCDD/Fs group (17.6 pg TEQ/g lipid).

## 3. Results

Nine articles published by our group on neurodevelopment in infants and children of various ages from the Da Nang cohort [4,5,6,7,8,9,10,11,12] are presented, with the age and sex of the subjects and evaluation methods in Table 1 for studies from 4 months to 3 years of age and in Table 2 for studies from 5 to 8 years of age.

To evaluate the general neurodevelopmental status of infants and children, standardized test batteries suitable for their age were used in each study (Table 1 and Table 2). To assess cognitive abilities, language, and motor development in infants and children, the Bayley Scales of Infant and Toddler Development, third edition (Bayley III) was used when the infants were approximately 4 months, 1 year, and 3 years of age. At 5 years of age, cognitive ability was assessed with the KABC-II, and coordination motor ability was examined with the MABC-2. For children aged 8 years, school performance related to their learning ability was evaluated using achievement tests for mathematics and Vietnamese, and with a reading test of a passage of a Vietnamese story.

Parent rating scales for several neurodevelopmental disorders were also used in these studies. To assess behaviors associated with autism spectrum disorder (ASD), the Autism Spectrum Rating Scale (ASRS; Multi Health Systems Inc., North Tonawanda, NY, USA) was used for children 3 and 5 years of age. In 5- and 8-year-old children, symptoms of attention deficit hyperactivity disorder (ADHD) were evaluated using the ADHD Rating Scale (ADHD-RS). The Colorado Learning Difficulties Questionnaire (CLDQ) [22] was used to assess the risk for learning difficulty at 8 years of age.

### 3.1. Associations between Dioxin Exposure Markers and Neurodevelopment in Children Aged 4 Months to 3 Years of Age (Table 1)

In infants 4 months of age in the Da Nang cohort, the fine motor scores were significantly lower in the high TEQ-PCDD/Fs exposure group (≥17.6 pg TEQ/g lipid). Increased levels of 1,2,3,7,8-pentachloro-dibenzo-p-dioxin (1,2,3,7,8-PeCDD), 1,2,3,7,8,9-hexa-chlorodibenzo-p-dioxin (HxCDD), and 1,2,3,4,6,7,8-hepta-chlorodibenzo-p-dioxin (1,2,3,4,6,7,8-HpCDD), which contributed to the elevated TEQ-PCDD/F levels, were also significantly associated with reduced fine motor scores, suggesting that PCDD congeners other than TCDD may influence infant neurodevelopment [5]. In a stratified analysis according to infant gender at the same age, only boys with high TEQ-PCDD/Fs (≥17.6 pg TEQ/g lipid) showed significantly lower Bayley III scores for expressive language development [6]. However, no increased Bayley III scores were observed in the high TCDD group (≥3.5 pg/g lipid) in either sex.

When the Da Nang birth cohort reached 1 year of age, we examined their neuro-development using the Bayley III scale again. However, no significant alterations in cognition, language, or motor scale scores associated with TEQ-PCDD/F levels were found in children of either sex, whereas lower social–emotional scale scores were observed in both the high TCDD group (≥3.5 pg/g lipid) and the high PCDD/Fs group (≥17.6 pg TEQ/g lipid) [7].

At 3 years of age, boys with higher TEQ-PCDD/Fs (≥17.6 pg TEQ/g lipid) showed lower scores in all domains, except for the fine motor scale, compared to the low-exposure group (<17.6). However, no difference was observed in girls [4]. The high TCDD group (≥3.5 pg/g lipid) showed high total and DSM-IV-TR Scale (DSM) scores in the ASRS, suggesting increased autistic traits, compared to the low exposure group.

Tai et al. (2016) [8] examined children using the Bayley III scale at 4 months, 1 year, and 3 years of age and analyzed all results longitudinally. These investigators found that boys with high TEQ-PCDD/Fs (≥17.6 pg TEQ/g lipid) showed lower marginal means of expressive language scores, and boys with high TCDD (≥3.5 pg/g lipid) showed lower marginal means of motor scores, particularly gross motor scores. However, no difference in any neurodevelopmental score associated with TEQ-PCDD/F or TCDD level was found in girls.

Taken together, the findings suggest that perinatal exposure to TEQ-PCDD/Fs of ≥17.6 pg TEQ/g lipid may influence neurodevelopment, particularly in boys in the first 3 years of life, although affected ability and skills may differ according to age. Furthermore, TCDD exposure of ≥3.5 pg/g lipid may increase autistic traits in both sexes, but its effect on general neurodevelopment may be limited and differ from the effects of other PCDD/F congeners.

### 3.2. Associations between Dioxin Exposure Markers and Neurodevelopment in Children Aged 5–8 Years of Age (Table 2)

At 5 years of age, only boys showed low non-verbal index (NVI) scores in the KABC-II, indicating cognitive deficit in the high TCDD group (≥2.5 pg/g lipid) [9]. Increased ADHD Rating Scale scores without increased ASRS scores were found in the high TCDD group (≥3.0 pg/g lipid) in boys [10]. In contrast, girls in the high TCDD group showed higher unusual behavior scores, an ASRS subscale score, although no increase in the ADHD Rating Scale score was found [10]. In addition, in boys with high TEQ-PCDD/Fs (≥17.6 pg TEQ/g lipid), MABC-2 scores, an index of coordination motor skills, were significantly lower; however, the NVI, ADHD, and ASRS rating scale scores were not associated with TEQ-PCDD/Fs levels [9,10].

At 8 years of age, boys with high TCDD (≥3.5 pg/g lipid) showed increased reading scores in the CLDQ, indicating a reading learning disability, such as dyslexia. They also showed significantly lower language achievement scores and higher reading errors in the reading test compared to boys with lower TCDD [1]. Boys with high 1,2,3,4,6,7,8-HpCDD (≥10.0 pg/g lipid) also showed higher CLDQ reading scores (more difficult), lower reading speed, and higher reading errors. Boys with high 1,2,3,4,7,8-HxCDD showed lower reading speed and higher reading errors, while the high TEQ-PCDD/Fs group (≥17.6 pg TEQ/g lipid) displayed higher reading errors [11]. These results suggest that reading disability, a neurodevelopmental disorder, might be prevalent in boys with high TCDD and 1,2,3,4,6,7,8-HpCDD levels. In addition, the high 1,2,3,4,7,8-HxCDD group and high HpCDD group had lower mathematics achievement test scores, suggesting these boys may have mathematics learning impairment as well as language learning deficits.

Among girls 8 years of age, the high TCDD (≥3.0 pg/g lipid) and high TEQ-PCDD/Fs (≥17.6 pg TEQ/g lipid) groups displayed higher hyperactivity scores, suggesting increased ADHD traits [10]. However, no association was found between learning ability, assessed with the CLDQ, and the reading test in girls.

Thao et al. (2020) [12] investigated sexual dimorphism in gaze behavior in 8-year-old children and reported that feminine index scores, defined as longer fixation duration on girl-oriented pictures, compared to boy-oriented pictures, were significantly higher in boys with high TEQ-PCDD/Fs (≥17.6 pg TEQ/g lipid), for almost all PCDD congeners. In girls, however, the feminine index scores were significantly higher only in the high TCDD group (≥3.5 pg/g lipid).

Taken together, TCDD exposure may be associated with the occurrence of neurodevelopmental disorders, such as ASD, ADHD, and learning difficulties (LD). In boys, however, not only TCDD exposure but also PCDD congeners, such as 1,2,3,4,6,7,8-HpCDD and 1,2,3,4,7,8-HxCDD, may influence cognitive ability and reading ability and change their behavior. However, in girls, only high TCDD (≥3.5 pg/g lipid) may contribute to the occurrence of adverse effects on their brain functions and behavior.

Taken together, the findings suggest that TCDD exposure may be associated with neurodevelopmental disorders, such as ASD, ADHD, and learning difficulties. In boys, however, not only exposure to TCDD, but also exposure to PCDD congeners, such as 1,2,3,4,6,7,8-HpCDD and 1,2,3,4,7,8-HxCDD, may affect cognitive and reading abilities and modify behavior. In comparison, in girls, only those with high TCDD (≥3.5 pg/g lipid) may exhibit changes in brain functions and behavior.

## 4. Discussion

### 4.1. Neurodevelopmental Disorders and Perinatal TCDD Exposure

We followed up perinatally dioxin-exposed infants from the Da Nang birth cohort for 8 years and found that exposure to TEQ-PCDD/Fs influenced language and motor development, particularly in boys, in the first 3 years of life. At 5 years of age, poor motor skills were also observed in boys with high TEQ-PCDD/Fs. Notably, TCDD exposure was specifically associated with an increased occurrence of neurodevelopmental disorders without intellectual disability, such as ASD, ADHD, or specific learning disorders (LDs). However, 1,2,3,4,7,8-HxCDD and 1,2,3,4,6,7,8-HpCDD, which are TEQ-PCDD/F constituents, were also associated with reading LDs in boys.

#### 4.1.1. ASD and Perinatal TCDD Exposure

ASD is a neurodevelopmental disorder that can be diagnosed around 2 years of age. However, previous studies in children exposed to high levels of dioxins and PCBs in the Netherlands [2] and Taiwan [23,24] have reported associations of dioxin exposure with general neurodevelopment, but not neurodevelopmental disorders such as ASD. Because neurodevelopmental disorder in children was only categorized as a psychiatric disorder approximately 20 years ago, studies published before 2000 did not examine associations with disorders such as ASD in children. Furthermore, children with increased ASD risk cannot be screened using general neurodevelopmental test batteries such as Bayley III, which were developed for early detection in children with an intellectual disability.

In the Da Nang cohort survey, we used ASRS to evaluate ASD behavior based on criteria in the Diagnostic and Statistical Manual for Mental Disorders, 4th Edition, text revision (DSM-IV-TR), published in 2009. This scale has three subscales, including social communication and unusual behavior. The symptoms and T-scores for each scale are calculated from raw values after percentile rank conversion, and it is a sensitive tool for screening children with autistic traits.

We found that children with TCDD levels ≥3.5 pg/g lipid showed significantly higher scores in the ASRS scales at 3 years of age compared to children with low exposure, in both sexes [4]. However, at 5 years of age, unusual behavior scores were significantly higher only in girls with TCDD ≥ 3.0 pg/g lipid [10]. A reason why boys showed no significant association with TCDD at 5 years of age might be the smaller number of children with high TCDD (≥3.5 pg/g lipid) in the survey compared to 3-year-olds. Another reason might be changes in behavior in children with high TCDD because of increased ADHD symptoms, particularly hyperactivity, in boys at 5 years of age with high TCDD ≥ 3.0 (pg/g lipid) [10]. This speculation is consistent with the significantly lower cognitive ability, assessed with the NVI in KABC-II, in boys with high TCDD (≥3.5 pg/g lipid), shown in Table 3, as well as high TCDD (≥2.5 pg/g lipid) [9]. In contrast, girls at 5 years of age did not show increased ADHD symptoms or lower NVI scores associated with high TCDD exposure.

#### 4.1.2. ADHD and Perinatal TCDD Exposure

ADHD is another neurodevelopmental disorder, and children with ASD are often diagnosed with ADHD as a comorbidity of ASD [25], and associations with exposure to endocrine disrupter chemicals, including PCB [26,27,28,29], pesticides [30,31], and dioxins [19,32], have been investigated in school-aged children. We also investigated associations between perinatal dioxin exposure and ADHD behaviors in children from the Da Nang cohort when they reached 8 years of age. Increased ADHD symptoms, particularly hyperactivity behaviors, were found in girls with TCDD ≥ 3.0 pg/g lipid or TEQ-PCDD/Fs ≥ 17.6 pg TEQ/g lipid, although they did not show increased ADHD symptoms at 5 years of age [10]. However, no increased aggressive behavior or ADHD associated with dioxin exposure was found in boys at 8 years of age, whose hyperactivity scores in the ADHD-RS were associated with TCDD exposure at 5 years of age.

Because it has often been observed in children with ADHD, aggressive behavior was also examined using the Children’s Scale of Hostility and Aggression: Reactive/Proactive (C-SHARP) with five subscales (verbal aggression, bullying, covert aggression, hostility, and physical aggression). The prevalence of high covert aggression scores in children, particularly in girls, was significantly higher in the high TCDD group (≥3.0 pg/g lipid) [33], suggesting behavior problems related to ADHD in girls exposed to high levels of TCDD.

#### 4.1.3. Learning Disorders (LDs) and Perinatal TCDD Exposure

Specific LD is a neurodevelopmental disorder that often co-occurs with other behavioral disorders in school-aged children [34,35]. CLDQ, which has two subscales—a math score and a reading score—is a good indicator of learning disability in school children.

An increased prevalence of LDs, based on interviewing parents, has been reported in school children exposed to background levels of dioxins and is associated with elevated serum levels of PCDDs and PCDF congeners [36]. Pham-The et al. (2020) [11] investigated the effects of perinatal dioxin exposure on learning difficulty using CLDQ and mathematics and language (Vietnamese) achievement tests in children from the Da Nang cohort when they reached school age. Boys with high TCDD (>3.5 pg/g lipid) or high 1,2,3,4,6,7,8-HpCDD (≥10.0 pg/g lipid) showed increased CLDQ reading scores, indicating a reading disability, such as dyslexia. Boys with high TCDD also showed significantly lower language achievement scores and higher errors and lower speed in reading a passage. High HpCDD and 1,2,3,4,7,8-HxCDD influenced not only reading learning but also mathematics learning, indicated by lower scores in mathematics achievement tests. These results suggest that TCDD exposure may mainly impair reading learning processes, including language processing, working memory, and processing speed control, which are collectively termed “lexical strategy”. In contrast, PCDD/F congeners other than TCDD, such as 1,2,3,4,7,8-HxCDD and 1,2,3,4,6,7,8-HpCDD, may affect the learning process of both mathematics and language in boys, suggesting different brain areas may be affected by these compounds.

#### 4.1.4. Gaze Behavior and Perinatal TCDD Exposure

Previous follow-up studies of children from birth cohorts in Duisburg, Germany [18] investigated the effects of total dioxin exposure on sex-typed play behavior in preschool children. These studies found increased feminine play behavior in boys and decreased masculine play behavior in girls, suggesting a feminization effect of dioxins, as endocrine disrupter chemicals. Thao et al. (2020) [12] examined gaze behavior in children at 8 years of age when viewing human line drawings, and found that feminine gaze behavior (preference for girl-oriented pictures) was increased in boys with high TEQ-PCDD/Fs (≥17.6 pg-TEQ/g lipid) and in girls with high TCDD (≥3.5 pg/g lipid). These investigators also examined salivary testosterone levels, an indicator of pubertal stage, in these children; however, an association of testosterone level with feminine gaze behavior was not found in either sex.

In boys, increased feminine gaze behavior, correlated with TEQ-PCDD/Fs, was found to be inversely correlated with hand movement scores and sequential index scores in the KABC-II examined at 5 years of age [9]. These findings suggest that impaired cognitive function, caused by dioxins perturbing brain development during the perinatal period, may lead to increased feminine gaze behavior in boys. In this study, girls with high TCDD (≥3.5 pg/g lipid) preferred female-oriented pictures, regardless of whether they were human or non-human, indicating an increased duration of fixed gaze behavior towards preferred pictures. This is often observed in children with ASD without an intellectual disability [37]. Moreover, in girls, lower cognitive scores related to facial recognition in the KABC-II examined at 5 years of age were associated with increased feminine gaze behavior observed at 8 years of age, suggesting poor facial recognition ability, which is often observed in children with ASD [38,39] in girls with high TCDD. These findings suggest that in girls, TCDD exposure may affect perinatal brain development and impair cognitive ability associated with social–emotional behavior, distinct from the cognitive functions affected in boys with high levels of TEQ-PCDD/Fs. Further studies are needed on the sex-specific effects of dioxins on social–emotional behavior during the adolescent period, when behaviors change drastically in both boys and girls.

### 4.2. Neurological Effects of Perinatal TCDD Exposure Detected by Electroencephalography (EEG)

In children 9 years of age in the Da Nang birth cohort, we investigated the effects of perinatal dioxin exposure on mu and theta rhythms by analyzing EEG recordings during hand movements, which reflect the activity of the mirror neuron system in the brain [40]. The mirror neuron system is reported to be impaired in children with ASD, resulting in poor social cognition. This concept is commonly called the “broken mirror theory” of autism [41]. In the studies in young (2–8-year-old) children, EEG power reduction in the theta band (4–8 Hz) during action observation or execution is a good indicator of mirror neuron activity [42]. In the Da Nang cohort, reduction in EEG power in the theta band caused by mirror neuron activity was significantly less in girls with high TCDD (≥3 pg/g lipid) and in boys with high TEQ-PCDD/Fs (≥17.6 pg TEQ/g lipid), particularly high HxCDDs and several PCDF congeners [40]. These results suggest that TCDD may be a congener that specifically impairs the mirror neuron system in the brain, resulting in social behavior problems, such as ADHD, in girls at 8 years of age [10]. However, in boys, not only TCDD, but also other PCDD/F congeners, may impact the neural substrates of cognitive functions, including the mirror neuron system, resulting in learning difficulties during school age.

Vu et al. (2021b) [43] performed magnetic resonance imaging (MRI) analysis in 32 men living in the most dioxin-contaminated area, originating from Agent Orange use near Bien Hoa Air Base in Vietnam, to investigate associations between dioxin exposure and brain structural irregularities. The volume of the left inferior frontal gyrus pars orbitalis, which participates in cognitive and social–emotional functions, was significantly lower in men exposed to Agent Orange, mainly TCDD, during the perinatal period. This suggests that TCDD may affect brain regions, leading to social cognitive deficits in men. In future studies, MRI imaging analysis should be performed in women in Bien Hoa to clarify the effects of TCDD on brain regions and on connectivity among different brain areas.

### 4.3. Estimated Threshold Values of TCDD for Significant Neurodevelopmental Problems

In the studies on children from the Da Nang cohort, the dioxin levels in breast milk were used as perinatal exposure markers, and cut-off values were calculated from the GM and GSD values in the breast milk of nursing mothers in unsprayed areas—3.5 pg/g lipid for the high TCDD group (GM × GSD^3^) and 17.6 (pg TEQ/g lipid) for the high TEQ-PCDD/Fs group (GM × GSD^4^) [5]. The effects of TCDD on KABC-II scores at 5 years of age and on ADHD-RS scores at 8 years of age were observed in children with TCDD ≥ 2.5 pg/g lipid [9] and TCDD ≥ 3.0 pg/g lipid [10], respectively, whereas the effects of TEQ-PCDD/Fs were only detected in children with TEQ-PCDD/Fs ≥ 17.6 pg TEQ/g lipid. These results suggest that the threshold value for TEQ-PCDD/Fs is approximately 18 pg TEQ/g lipid. In comparison, the estimation of TCDD threshold values is difficult.

In girls, neurotoxic effects appeared after 5 years of age and were specific to TCDD exposure [10]. Furthermore, the proportion of children with high TCDD levels was higher among girls compared to boys at different cut-off values, including 2.5–5.5 pg/g lipid (Table 3). Particularly, when the cut-off value for the high TCDD group was set at 5.5 pg/g lipid, the proportion of all children with high TCDD was significantly greater in girls compared to boys (*p* = 0.035). These findings suggest that more girls were exposed to extremely high levels of TCDD during the fetal period compared to boys, resulting in a higher frequency of TCDD-specific neurotoxic effects in girls. Interestingly, a lower sex ratio at birth (lower percentage of boys), associated with serum TCDD levels, was reported in residents exposed to high levels of TCDD following a chemical factory explosion in Seveso, Italy [44], suggesting that female fetuses are relatively resistant to TCDD toxicity compared to male fetuses.

Taken together, the findings suggest that girls exposed to high levels of TCDD may be able to grow up without neurodevelopmental problems, except social–emotional cognitive deficits, similar to children with ASD and ADHD without intellectual deficits. However, the number of children with high TCDD exposure is too small to estimate sex-specific threshold values for the neurotoxic effects of TCDD. Future studies on children from the Bien Hoa cohort, including more children exposed to high TCDD levels (≥5.5 pg/g lipid), may help address this shortcoming [45].

### 4.4. TCDD-Induced Neurotoxicity via the Aryl Hydrocarbon Receptor (AhR)

Most, if not all, of the toxic and biological effects of TCDD are mediated through the aryl hydrocarbon receptor (AhR), as are the effects of PCDD/F congeners [21]. Thus, the total TEQ-PCDD/F value is frequently used to estimate the total TCDD-like toxicity of all PCDD/Fs. Notably, however, our findings suggest that only TCDD increased the risk for ASD in 3-year-old children [4], LD in boys [11], and ADHD in girls at 8 years of age [10] after adjusting for confounding factors, such as maternal age, education, smoking of family members, drinking habit during pregnancy, and economic status.

As perinatal dioxin exposure markers, dioxins in maternal breast milk were used in the Da Nang birth cohort studies, of which relevant factors to each congener were investigated by Tai et al. (2011) [1] and Anh et al. (2014) [46]. The length of residency nearby the air base was the most important factor in increased dioxin levels in breast milk, although some food consumption increased several PCDD/Fs congeners. Particularly, TCDD concentrations were highly associated with only increased residency around Da Nang Air Base, suggesting that TCDD exposure may have originated from contamination of Agent Orange in the soil and sediment around Da Nang Air Base. 

Marazziti et al. (2012) [47] reviewed the studies to investigate associations between mitochondrial alterations and neuropsychiatric diseases, including neurodevelopmental disorders, and suggested that mitochondrial abnormalities may have a role in the onset or pathophysiology in developmental disorders, such as autism and ADHD. Additionally, in a recent clinical study, Lee et al. (2019) [48] reported that only girls with ADHD showed higher levels of HtrA2 in plasma, a mitochondria-associated protein, compared to controls, and that their HtrA2 levels were inversely correlated with behavioral symptoms. These findings indicate that mitochondrial pathways may have an important role in the pathophysiology of ADHD in girls.

In an in vitro study, Hwang et al. (2016) [49] reported that a portion of the cellular pool of AhR was localized to the inter-membrane space of the mitochondria and that TCDD exposure induced degradation of the AhR pool in mitochondria, resulting in altered cellular respiration and influencing a battery of proteins associated with various metabolic pathways within the mitochondrial proteome. These results suggest that TCDD exposure may induce mitochondrial dysfunction via AhR, leading to cell energy metabolic abnormality.

Taken together, and compared to other PCDD congeners, TCDD might have more specific AhR-dependent toxicity to induce mitochondrial dysfunction, which is involved in the pathophysiology of neurodevelopmental disorders, such as ASD and ADHD, observed in children from the Da Nang cohort. Further studies are required to clarify the differences in neurotoxic effects between TCDD and other PCDD/Fs congeners and their mechanisms in the future.

## 5. Conclusions

Perinatal TCDD exposure affects social–emotional cognitive functions, leading to neurodevelopmental disorders. However, the effects are sex-specific: primarily LD in boys and predominantly ADHD in girls. PCDD/F congeners other than TCDD may specifically impact neurodevelopment in boys. The estimated threshold values for TCDD may also differ between the sexes. Further studies on children, including those with high TCDD exposure, are necessary to clarify the sex and age-specific neurodevelopmental effects of these dioxins.

## Figures and Tables

**Table 1:** Neurodevelopment and perinatal dioxin exposure in infants and children aged 4 months to 3 years.

Authors	Age	Neurodevelopmental Markers (Test Battery/Scale)	Results
Tai (Pham-The) et al. [5]	4 months *N* = 210	Cognitive, language, motor scale scores (Bayley III)	Infants with high TEQ-PCDD/Fs (≥17.6) showed lower cognitive and fine motor scores. Infants with moderately higher TCDD (1.8–3.5) showed lower scores for all domains. The increased scores in infants with TCDD ≥ 3.5 were not significant.
Nishijo M et al. [6]	4 months *N* = 210	Cognitive, language, motor scale scores (Bayley III)	Only boys showed lower expressive language scores in those with high TEQ-PCDD/Fs (≥17.6).
Pham TT (Pham-The) et al. [7]	1 year *N* = 214	Cognitive, language, motor, social–emotional, adaptive behavior scale scores (Bayley III)	The high TCDD group (≥3.5) and high TEQ-PCDD/Fs group (≥17.6) showed lower social–emotional scale scores. However, no difference in other developmental scale scores was found among the other groups.
Nishijo M et al. [4]	3 years *N* = 198	Cognitive, language, motor, social–emotional, adaptive behavior scale scales (Bayley III)	Boys with high TEQ-PCDD/Fs (≥17.6) showed lower scores in all domains, except the fine motor scale, compared to the lower group (<17.6). However, no difference was observed in girls.
		Total score (TOT), DSM-IV-TR Scale (DSM), social communication (SC), unusual behavior (UB) scores (ASRS)	In both sexes, those with high TCDD (≥3.5) showed high TOT and DSM scores (increased autistic traits) compared to the low exposure group.
Tai (Pham-The) et al. [8]	4 months to 3 years *N* = 217	Cognitive, language, motor scale scores (Bayley III)	Among boys, the high TCDD group (≥3.5) showed lower marginal means of composite motor scores and gross motor scores. The high TEQ-PCDD/Fs (≥17.6) group showed lower marginal means of expressive language scores. However, no differences in any scales were found among girls.

Units: pg/g lipid for TCDD and pg TEQ/g lipid for TEQ-PCDD/Fs, *N*: number of subjects, Bayley-III: Bayley Scales of Infant and Toddler Development, 3rd edition; ASRS: Autism Spectrum Rating Scale.

**Table 2:** Neurodevelopment and perinatal dioxin exposure in children aged 5–8 years.

Authors	Age	Neurodevelopmental Markers (Test Battery/Scale)	Results
Tran NN et al. [9]	5 years, *N* = 181	Non-verbal index for cognitive functions (NVI), sequence/general ability of short-term memory (Seq/GSM), simultaneous/general ability of visual processing (Sim/GV) scores (KABC-II)	Only boys with high TCDD (≥2.5) showed lower scores in the NVI and pattern reasoning scores, an NVI component, compared to boys with lower TCDD (<2.5). However, no association with TEQ-PCDD/Fs was found in either sex.
		Total scale for coordination movement skills (TOTAL), manual dexterity (MD), aiming and catching (A&C), balance (BAL) scores (MABC-2)	In boys only, the high TEQ-PCDD/Fs group (≥17.6) showed lower TOTAL and BAL scores compared to the low (<11.5) and moderate (11.5–17.6) exposure groups. However, no association with TCDD was found in either sex.
Pham-The et al. [10]	5 years, *N* = 163	Total scale of ADHD symptoms (ADHD), inattention scale (Inattention), impulsivity and hyperactivity scale (Hyperactivity) scores (ADHD-RS)	Boys with high TCDD (≥ 3.0) showed higher hyperactivity and ADHD scores. However, no association was found with TEQ-PCDD/Fs (≥ 17.6) in boys and with either dioxin marker in girls.
		Total score (TOT), DSM-IV-TR Scale (DSM), social communication (SC), unusual behavior (UB) scores (ASRS)	In girls, those with high TCDD (≥3.0) showed higher UB scores. However, there was no association between any ASRS index and exposure markers in boys.
	8 years, *N* = 163	Total scale of ADHD symptoms (ADHD), inattention scale (Inattention), impulsivity and hyperactivity scale (Hyperactivity) scores (ADHD-RS)	Girls with high TCDD (≥3.0) or high TEQ-PCDD/Fs (>17.6) showed higher hyperactivity scores. However, no difference in any ADHD scores between high and low exposure groups in boys.
Pham-The et al. [11]	8 years, *N* = 185	CLDQ reading, CLDQ math scores (CLDQ)	In boys only, those with higher TCDD (≥3.5) and higher 1,2,3,4,6,7,8-HpCDD (>10.0) showed higher CLDQ reading scores compared to the lower exposure groups.
		Vietnamese and mathematics test scores (Achievement tests)	In boys, the high TCDD (≥3.5) group showed low language achievement scores and the high 1,2,3,4,7,8-HxCDD group showed lower mathematics and language scores. Those with high 1,2,3,4,6,7,8-HpCDD showed lower mathematics scores.
		Speed and number of errors for reading a short Vietnamese passage (Reading test)	In boys, high TCDD (≥3.5) group showed higher reading errors. The high TEQ-PCDD/Fs group (≥17.6) showed higher reading errors. The high 1,2,3,4,7,8-HxCDD and 1,2,3,4,6,7,8-HpCDD groups showed lower reading speed and higher reading errors.
Thao P.N. et al. [12]	8 years, *N* = 172	Duration of fixed gaze behavior on the picture (Eye tracking)	High TCDD exposure (≥3.5) increased the feminine index for viewing human line drawings in girls. Boys with high TEQ-PCDD/Fs (≥17.6) displayed a high feminine index. Almost all PCDD congeners were associated with an increased index in boys.

Units: pg/g lipid for TCDD and pg TEQ/g lipid for TEQ-PCDD/Fs, ASRS: Autism Spectrum Rating Scale; ADHD-RS: Attention Deficit Hyperactivity Disorder Rating Scale, CLDQ: Colorado Learning Difficulty Questionnaire.

**Table 3:** Differences in toxin levels between sexes in children with high TCDD for each TEQ-PCDD/F category.

	TEQ-PCDD/Fs														
	<17.6					>17.6					All				
	Boys *N* = 104		Girls *N* = 73		*p*	Boys *N* = 24		Girls *N* = 26		*p*	Boys *N* = 128		Girls *N* = 99		*p*
Mean, SD of TEQ	10.6	1.4	10.5	1.5	0.826	22.5	1.2	23.3	1.3	0.620	12.2	1.6	12.9	1.7	0.392
Mean, SD of TCDD	1.2	2.0	1.1	2.3	0.749	2.6	1.7	3.2	1.9	0.236	1.3	2.1	1.5	2.5	0.430
*N*, (%) of TCDD > 2.5	12	(11.5)	8	(11.0)	0.904	10	(41.7)	18	(69.2)	0.050	22	(17.2)	26	(26.3)	0.098
*N*, (%) of TCDD > 3.0	5	(4.8)	4	(5.5)	0.842	7	(29.2)	11	(42.3)	0.332	12	(9.4)	15	(15.2)	0.184
*N*, (%) of TCDD > 3.5	3	(2.9)	3	(4.1)	0.660	5	(20.8)	10	(38.5)	0.171	8	(6.3)	13	(13.1)	0.086
*N*, (%) of TCDD > 5.5	0	(0.0)	0	(0.0)	-	2	(8.3)	7	(26.9)	0.079	2	(1.6)	7	(7.1)	0.035

Units: pg/g lipid for TCDD and pg-TEQ/g lipid for TEQ-PCDD/Fs, *N*: number of subjects, SD: standard deviation; TEQ: TEQ-PCDD/Fs, *p*: *p*-values compared between boys and girls by likelihood ratio test.

## Data Availability

Not applicable.
